# Effects of dietary supplementation with rice bran oil on the growth performance, blood parameters, and immune response of broiler chickens

**DOI:** 10.1186/s40781-016-0092-6

**Published:** 2016-03-15

**Authors:** Hwan Ku Kang, Chan Ho Kim

**Affiliations:** Poultry Science Division, National Institute of Animal Science, RDA, Seonghwan-eup, Cheonan-si, Chungcheognam-do 330-801 Republic of Korea

**Keywords:** Broiler, Growth performance, Rice bran oil, Total cholesterol

## Abstract

**Background:**

The objective of this experiment was to investigate the effects of dietary supplementation of rice bran oil (RBO) on growth performance, blood parameter, and immune response in broiler chickens.

**Methods:**

A total of 240 1-d-old ROSS 308 male broilers were randomly allotted to 4 dietary treatments with six replicated pens consisting of ten chicks. The basal diet was formulated to be adequate in energy and nutrients. Three additional diets were prepared by adding 5, 10 or 20 g/kg of RBO to the basal diet. The experimental diets were fed on an *ad libitum* basis to the birds during 35 d.

**Results:**

Results indicated that increasing inclusion level of RBO in diets improved BW gain (linear and quadratic, *P* < 0.01), improve feed conversion ratio (linear, *P* < 0.05) of birds during 0 to 35 d. There was no effect of inclusion level of RBO in diets on feed intake of birds. There was no effect of inclusion level of RBO in diets on erythrocytes of birds. However, heterophil, lymphocyte, and monocytes increased (linear and quadratic, *P* < 0.01) with inclusion level of RBO in diets increased. Feeding the diets containing increasing amount of RBO to birds increased (linear, *P* < 0.01) the concentrations of total cholesterol. Increasing inclusion level of RBO in diets increased concentrations of IgG (linear, *P* < 0.01). There was no effect of inclusion level of RBO in diets on concentrations of IgM.

**Conclusions:**

These results suggest that dietary RBO may be used functional ingredient to improve growth performance, total cholesterol in serum, and immune response of birds.

## Background

Currently, consumers are increasingly aware of the health benefits and nutritional quality of the food they consume [1]. Rice is the principle cereal food in Korea, and the by-products from its milling are used as important feed resources. Rice bran, a by-product of rice milling, contains high levels of important nutrients, such as proteins, vitamins, minerals, complex carbohydrates, phytonutrients, phospholipids and essential fatty acids [2–4]. Rice bran is an effective dietary energy and unsaturated fatty acid source for animals such as chickens, rats and pigs with relatively minor effects on growth and performance [5]. One such by-product is rice bran oil (RBO); it is gaining commercial importance worldwide because of its many beneficial nutritive and biological effects. Rice bran oils contains 90–96 % saponifiable lipids and about 4 % unsaponifiable lipids. The saponifiable lipids include 68–71 % triglycerides, 2–3 % diglycerides, 5–6 % monoglycerides, 2–3 % free fatty acids (FFAs), 2–3 % waxes, 5–7 % glycolipids, and 3–4 % phospholipids [6], whereas the principle component of the unsaponifiable fraction is ɤ-oryzanol [7]. RBO can be extracted from rice bran by using solvent extraction with food-grade *n*-hexane [8], via a solvent-free process by using ohmic heating [9], or by extraction using ethanol [10]. ɤ-oryzanol, a phytosterylferulate mixture extracted from rice bran oil, has a wide spectrum of biological activities, as well as antioxidant properties [11]. RBO has an excellent fatty acid profile. Three major fatty acids, palmitic, oleic, and linoleicaccount for 90 % of the total fatty acids of rice bran extracts [12]. Previous studies on dietary supplementation of rice bran oil in broilers have shown improved growth performance [13, 14] and decreased cholesterol concentrations [15]. Dietary supplementation of rice bran in mice have shown improved immune response [16]. However, data pertaining to the effects of dietary supplementation of RBOon the growth performance, blood parameters, and immune response of broilersare limited. Therefore, the objective of the present study was to investigate the effect of dietary supplementation of rice bran extracts on the growth performance, blood parameters, immune responseof broilers.

## Methods

The protocol for this experiment was reviewed and approved by the Institutional Animal Care and Welfare Committee of the National Institute of Animal Science, Rural Development Administration, Republic of Korea.

### Preparation of rice bran oil (RBO)

**Fig. 1:**
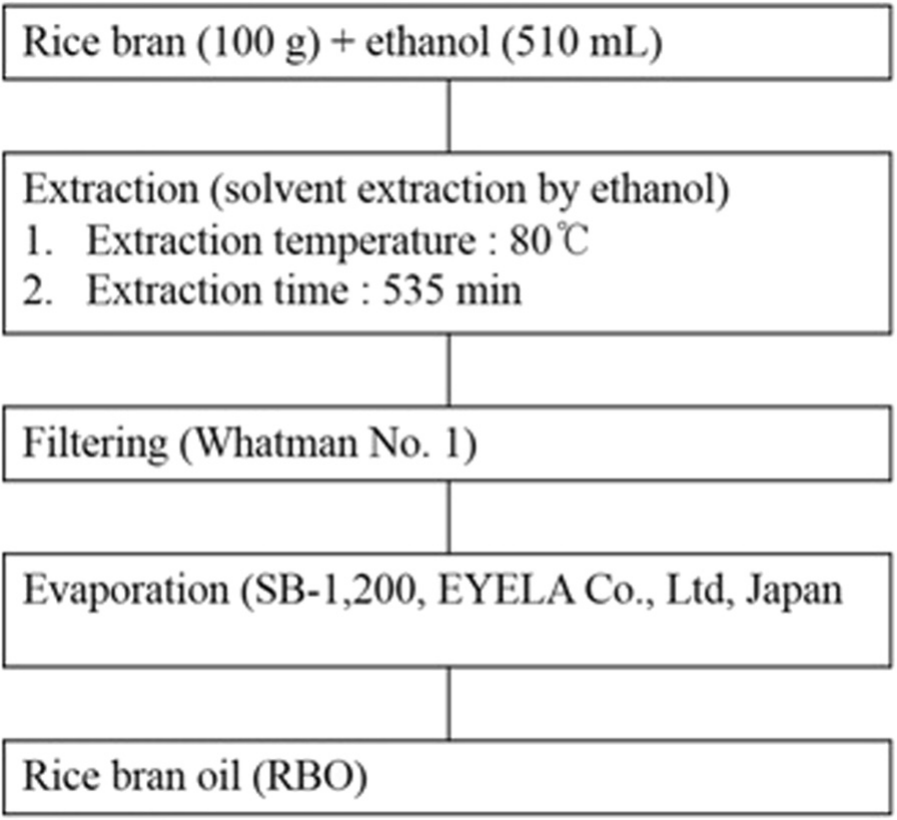
Scheme for extraction process from rice bran

Rice bran harvested in 2014 was obtained from a rice processing complex (Seonghwan-eup, South Korea). RBO was extracted as described previously ([10] Fig. 1). The nutrient composition of RBO was analyzed in duplicate for crude fat [17]. The results are presented in Table 1.

**Table 1:** Analyzed fatty acids composition of rice bran oil (RBO) used in this experiment (as is basis)

Composition	Rice bran oil (RBO)^a^
Crude fat (g/kg)	305.1
Fatty acids composition	g/kg
Myristic acid (C14:0)	3.6
Palmitic acid (C16:0)	179.6
Palmitoleic acid (C16:1n7)	2.1
Stearic acid (C18:0)	0.6
Vaccenicacid (C18:ln7)	13.1
Oleic acid (C18:1n9)	422.5
Linoleic acid (C18:2n6)	378.5
Total	1,000.0
Saturated fatty acid	183.8
Unsaturated fatty acid	816.2
Mono unsaturated fatty acid	433.7
Poly unsaturated fatty acid	378.5

^a^Nutrient composition was analyzed for crude fat (AOAC, 1995; method 920.39)

### Birds and experimental design

A total of 240 1-d-old ROSS 308 male broilers (initial BW = 36 ± 0.74 g) were obtained from local hatchery (Samhwa Hatchery, Hongseong, Republic of Korea) and were housed in a battery cages (86 cm × 57 cm × 35 cm, width × length × height) in an environmentally controlled room. The experiment was performed as a completely randomized design with six replicate pens consisting of ten chicks. A 2-phase feeding program with a starter diet from 0 to 21 d and a grower diet from 22 to 35 d was used in this experiment (Table 2). Within each phase, a control diet was formulated to meet or exceed NRC [18] requirements of broiler chickens for macro- and micronutrients. Three additional diets were prepared by adding 5,10 or 20 g/kg of RBO to the basal diet. The RBOwas added at the expense of the basal diet. The experimental diets were mash form. The diets and water were available*ad libitum*. The room temperature for both conventional and battery cages was maintained at 30 °C during the first week of the experiment and then gradually decreased to 24 °C at the end of the experiment as recommended by the Ross manual. A 24-h lighting schedule was used throughout the experiment.

**Table 2:** Composition and nutrient content of experimental diets (as-fed basis)

Item	Starter period (0 to 21 d)				Grower period (22 to 35 d)			
	Rice bran oil (g/kg)				Rice bran oil (g/kg)			
	0	5	10	20	0	5	10	20
Ingredients, g/kg								
Maize	509.1	504.1	499.1	489.1	598.9	593.9	588.9	578.9
Soybean meal (450 g/kg CP)	356.4	356.4	356.4	356.4	322.2	322.2	322.2	322.2
Corn gluten meal	35.0	35.0	35.0	35.0	-	-	-	-
Soybean oil	60.0	60.0	60.0	60.0	45.0	45.0	45.0	45.0
Dicalcium phosphate	18.6	18.6	18.6	18.6	13.5	13.5	13.5	13.5
Limestone	15.4	15.4	15.4	15.4	15.2	15.2	15.2	15.2
Methionine-99 %	1.5	1.5	1.5	1.5	1.2	1.2	1.2	1.2
NaCl	2.0	2.0	2.0	2.0	2.0	2.0	2.0	2.0
Vitamin-mineral premix^a^	1.0	1.0	1.0	1.0	1.0	1.0	1.0	1.0
Sodium bicarbonate	1.0	1.0	1.0	1.0	1.0	1.0	1.0	1.0
Rice bran oil	-	5.0	10.0	20.0	-	5.0	10.0	20.0
Total	1,000.0	1,000.0	1,000.0	1,000.0	1,000.0	1,000.0	1,000.0	1,000.0
Energy and nutrient content^b^								
ME_n_ (MJ/kg)	13.1	13.1	13.1	13.1	13.0	13.0	13.0	13.0
Crude protein (g/kg)	230.0	230.0	230.0	230.0	200.0	200.0	200.0	200.0
Calcium (g/kg)	10.0	10.0	10.0	10.0	9.0	9.0	9.0	9.0
Avaiable P (g/kg)	4.3	4.3	4.3	4.3	3.5	3.5	3.5	3.5
Lysine (g/kg)	11.9	11.9	11.9	11.9	10.8	10.8	10.8	10.8
Methione + cysteine (g/kg)	8.5	8.5	8.5	8.5	7.2	7.2	7.2	7.2

^a^Provided per kilogram of the complete diet: vitamin A (from vitamin A acetate), 12,500 IU; vitamin D_3_, 2,500 IU; vitamin E (from DL-α-tocopheryl acetate), 20 IU; vitamin K_3_, 2 mg; vitamin B_2_, 5 mg; vitamin B_6_, 3 mg; vitamin B_12_, 18 μg; calcium pantothenate, 8 mg; folic acid, 1 mg; biotin, 50 μg; niacin, 24 mg; Zn (as ZnO), 60 mg; Mn (as MnSO_2_ · H_2_O), 50 mg; Fe (as FeSO_4_ · 7H_2_O), 50 mg; Cu (as CuSO_4_ · 5H_2_O), 6 mg; Co (as CoCO3), 250 μg; I [as Ca(IO_3_)_2_ · H_2_O], 1 mg; Se (as Na_2_SeO_3_), 150 μg

^b^Nutrient contents in all diet were calculated were analyzed

### Growth performance

Body weight gain and feed intake were recorded at 21 and 35 d of the experiment. Feed conversion ratio was calculated as feed intake divided by BW gain.

### Sample collection and analysis

At the end of the 35-day feeding trial, 1 bird with a BW close to the pen BW (i.e. six birds) was selected and euthanized by cervical dislocation. Immediately after euthanasia, a 5 mL blood sample was collected from the jugular vein by using EDTA vacuum tubes (Becton Dickinson, Franklin Lakes, NJ), stored on ice, and subjected to immediate hematological analysis. Leukocytes (white blood cells, heterophils, lymphocytes, monocytes, eosinophils, basophils) and erythrocytes (red blood cells, hemoglobin, hematocrit, and mean corpuscular hemoglobin concentration) were quantified using Hemavet Multispecies Hematology Systems (Drew Scientific Inc., Oxford, CT). Immediately after the blood analysis, serum samples, obtained by centrifuging the samples for 20 min at 25,000 × *g* and 4 °C, were stored at –15 °C. Aspartate aminotransferase (AST), alanine aminotransferase (ALT), glucose, total cholesterol, and albumin in the serum were quantified using an ADVIA 1650 chemistry system (Bayer diagnostic, Putraux, France). The serum samples were used to measure the concentrations of IgG and IgM isotype, using chicken IgG or IgM ELISA quantitation kits (Bethyl Laboratories, Montgomery, TX). Briefly, flat bottomed microtiter plates were coated for 60 min with capture antibody (goat anti-chicken IgG or IgM affinity purified) and coating buffer (0.05 M carbonate-bicarbonate, pH 9.6). Plates were washed 3 times with washing solution (50 mMTris-buffered saline, 0.14 M NaCl, 0.05 % Tween 20, pH 8.0), and wells were incubated with blocking solution (50 mMTris-buffered saline, 0.14 M NaCl, 1 % BSA, pH 8.0) for 30 min then rinsed 3 times with washing solution. The calibrator (chicken reference plasma) and sample-conjuate diluent (50 mM Tris-buffered saline, 0.14 mM NaCl, 1 % BSA, 0.05 % Tween 20, pH 8.0) were used to do standards, whereas plasma samples, thawed at 4 °C overnight, were diluted at 1:1,000 in the sample-conjugate diluent. Then, they were incubated in wells for 60 min and washed 5 times with washing solution. Detection antibody horserasishperosidase (goat anti-chicken IgG or IgM) diluted in sample-conjugate diluent was added to wells, incubated for 60 min, and rinsed 5 times with washing solution. Enzyme substrate (3, 3’, 5, 5’-tetramethyl benzidine peroxidase substrate and peroxidase solution B) was added and incubated for 15 min (IgM) or 30 min (IgG). Finally, 2 M H_2_SO_4_ was used to stop the 3’, 3–5, 5’-tetramethyl benzidine reaction. A microtiter plate reader (Spectramax 190, Molecular Device, USA) was used to measure the absorbance at 450 nm. To calculate the immunoglobulin (IgG or IgM) concentration, a 4-parameter logistic curve fit was developed using the broiler chicken reference plasma absorbance.

### Statistical analysis

All data were analyzed by ANOVA according to completely randomized design [19] using the PROC MIXED procedure of SAS (SAS Institute Inc., Cary, NC). Outlier data were identified according to STEEL et al. [19], using the UNIVARIATE procedure of SAS, but no outliers were detected. The battery cage was an experimental unit for growth performance data, whereas the individual bird was an experimental unit for blood parameter, serum enzyme activities and immune response data. Dietary treatment was fixed effect in all statistical models. The LSMEANS procedure was used to calculate mean values. The orthogonal polynomial contrast test was performed to determine linear and quadratic effects of increasing inclusion level of RBO in diets on each measurement. Significance and tendency for statistical tests were set at *P* < 0.05 and 0.05 ≤ *P* ≤ 0.10, respectively.

## Results and discussion

During the initial 21 d of the experiment, BW gain and feed conversion ratio increased (linear, *P* < 0.05) with inclusion level of RBE, but feed intake was not influenced by inclusion of RBE in diets (Table 3). During 22 to 35 d of the experiment, BW gain increased (linear and quadratic, *P* < 0.05) with inclusion level of RBO and a tendency for improved feed conversion ratio (linear, *P* < 0.10) was observed as inclusion level of RBE in diets increased. There was no effect of inclusion level of RBO in diets on feed intake of birds. For overall experiment, increasing inclusion level of RBO in diets increased BW gain (linear and quadratic, *P* < 0.01), and improved feed conversion ratio (linear, *P* < 0.05). There was no effect of inclusion level of RBO in diets on feed intake of birds. Dietary supplementation of rice bran or rice bran extracts has been reported to improve BW gain in broilers [20, 21]. It is suggested that positive effects of dietary rice bran extracts on birds’ performance may result from its high concentrations of oryzanols, tocopherols, vitamin E, ferulic acid, phytic acid, lecithin, and inositol [10, 22]. Advantages of utilizing oils in broiler diet include increase in absorption and digestion of lipoproteins, significance amount of necessary fatty acids [23–25]. In addition, the favorable results of vegetable oil diet on growth performance of birds could be explained by the positive effect of this fat sources on the reduced passage rate of the digesta through the gastrointestinal trait, allowing for better nutrient absorption and utilization [26], resulting in a more efficient use of nutrients from diet. There was no effect of inclusion level of RBE in diets on erythrocytes of birds. However, heterophil, lymphocyte, and monocytes increased (linear and quadratic, *P* < 0.01) with inclusion level of RBE in diets increased (Table 4). Blood parameters are good indicators of physiological, pathological and nutritional status of an animal and changes in hematological parameters have the potential of being used to elucidate the impact of nutritional factors and additives supplied in diet on any living creature. For example, leukocytes are known to increase sharply when infection occurs, as they are one of the first lines of defense of the body [27–29]. However, the lack of adequate data on the role of rice bran extracts in altering blood parameters in poultry requires further research.As expected, asincreasing inclusion level of RBE in diets decreased (linear, *P* < 0.01) the concentrations of total cholesterol (Table 5). AST, ALT, glucose, and albumin were not affected by inclusion of RBO in diets. ANITHA et al. [13] reported that dietary supplementation of rice bran extracts decreased the concentrations of total serum cholesterol in broilers. Several studies on humans and animals [22, 30–32] showed that rice bran extracts lowered the level of low-density lipoprotein cholesterol and total serum cholesterol or enhancing the conversion of cholesterol to fecal bile acids and sterols. Levels of lipid serum (total cholesterol, beta lipoprotein, and LDL cholesterol) decreased significantly [33]. It was concluded that oryzanol was at least partly responsible for the cholesterol lowering action of RBE and is associated with the reduction in aortic fatty streak formation. ɤ-oryzanol can also lower the plasma cholesterol level [34]. Although the mechanism underlying this effect is not apparent at present the presence of oryzanol and tocopherols in the rice bran is thought to be responsible for this favorable effect. Nutritional and biochemical aspects of the hypolipidemic action of rice bran extracts have been reviewed by Rukmini and Raghuram [35]. Increasing inclusion level of RBE in diets increased concentrations of IgG (linear, *P* < 0.01). There was no effect of inclusion level of RBE in diets on concentrations of IgM (Table 6). RBO effect on the immune response has been suggested to be due to its adequate mixture of essential fatty acids, and unsaturated fatty acid. Yang et al. [36] reported that vegetable oil such as canola oil has been recognized as adequate mixture of essential fatty acids, and unsaturated fatty acid such a linolenic acid that can improve broiler performance, also linolenic acids can be converted to longer chain omega-3 fatty acid. The action of n-6 PUFA as a pro-inflammatory factor and the action of n-3 PUFA as an anti-inflammatory factor is well documented [37]. Dietary lipid source may affect immuno-competence via affecting membrane fatty acid composition and therefore fluidity, flexibility and function and affecting the inflammatory process and other cell signaling pathways [38]. Our result suggested immunomodulatory effects produced by the consumption of RBO, which also indicate that ɤ-oryzanol is not responsible for the overall immuno-regulation despite having important roles in other organism functions. In fact, ɤ-oryaznol modifies the immune response in a different way to the one observed for RBO. Thus, fatty acid composition could be responsible for the effects of RBO. A high concentrations linoleic acid consumption could be responsible for the increase in lymphocyte proliferation, spleen size and TH1 repsonse as suggested by other in vivo and in vitro studies using linoleic acid [39–41]. RBO contains an important fraction of linoleic acid, and the effects described in the above mentioned studies are also observed in our study.

**Table 3:** Growth performance of broiler chickens fed the diet containing rice bran oil (RBO)^a^

Item	Dietary treatments^b^				Pooled SEM^c^	*P-*value	
	0	5	10	20		Linear	Quadratic
0 to 21 d							
BW gain (g/bird)	720.5	763.4	769.2	774.5	13.52	0.02	0.20
Feed intake (g/bird)	1,095.8	1,033.1	1,008.3	985.4	49.62	0.14	0.70
Feed conversion ratio	1.50	1.35	1.31	1.27	0.07	0.02	0.34
22 to 35 d							
BW gain (g/bird)	835.4	931.8	907.7	895.6	14.91	0.04	<0.01
Feed intake (g/bird)	1,739.4	1,736.3	1,689.4	1,659.8	84.44	0.27	0.87
Feed conversion ratio	2.15	1.86	1.86	1.86	0.09	0.07	0.18
0 to 35 d							
BW gain (g/bird)	1,555.9	1,695.2	1,676.9	1,670.1	8.92	<0.01	<0.01
Feed intake (g/bird)	2,889.3	2,769.3	2,697.8	2,645.2	119.5	0.17	0.79
Feed conversion ratio	1.86	1.63	1.61	1.58	0.07	0.03	0.22

^a^Data are least squares means of 6 observations per treatments

^b^Basal diet was supplemented with RBO at 5, 10, or 20 g/kg

^c^Pooled standard error of the means

**Table 4:** Blood parameter of broiler chickens fed the diet containing rice bran oil (RBO)^a^

Items	Dietary treatments^b^				Pooled SEM^c^	*P-*value	
	0	5	10	20		Linear	Quadratic
Leukocytes^d^							
WBC (K/μL)	14.34	12.27	13.62	14.97	1.70	0.61	0.56
HE (K/μL)	0.28	2.15	2.23	2.06	0.30	<0.01	<0.01
LY (K/μL)	3.75	8.78	9.56	6.65	0.91	<0.01	<0.01
MO (K/μL)	0.30	1.14	1.35	0.91	0.13	<0.01	<0.01
EO (K/μL)	0.01	0.18	0.16	0.27	0.04	0.98	0.24
BA (K/μL)	0.00	0.03	0.02	0.04	0.02	0.18	0.25
Erythrocytes^e^							
RBC (M/μL)	2.48	2.54	2.61	2.27	0.09	0.65	0.20
Hb (g/dL)	9.83	10.03	10.07	9.40	0.26	0.76	0.25
HCT (%)	24.47	25.03	25.17	22.90	0.87	0.99	0.12
MCV (fL)	98.60	98.43	96.80	100.95	1.64	0.54	0.78
MCH (pg)	39.70	39.43	38.67	41.50	0.82	0.18	0.30
MCHC (g/dL)	40.23	40.10	40.00	41.05	0.79	0.46	0.36

^a^Data are least squares means of 6 observations per treatments

^b^Basal diet was supplemented with RBO at 5, 10, or 20 g/kg

^c^Pooled standard error of the means

^d^Leukocytes: *WBC* white blood cells, *HE* heterophils, *LY* lymphocytes; heterophil : lymphocytes; *MO* monocytes, *EO* eosinophils, *BA* basophils

^e^Erythrocytes: *RBC* red blood cells, *Hb* hemoglobin, *HCT* hematocrit, *MCV* mean corpuscular volume, *MCH* mean corpuscular hemoglobin, *MCHC* mean corpuscular hemoglobin concentration

**Table 5:** Serum enzyme activities of broiler chickens fed the diet containing rice bran oil (RBO)^a^

Items	Dietary treatments^b^				Pooled SEM^c^	*P*-value	
	0	5	10	20		Linear	Quadratic
AST(U/L)	188.0	188.7	192.7	208.0	12.68	0.43	0.14
ALT (U/L)	5.33	4.67	5.33	5.00	0.62	0.82	0.17
Total cholesterol (mg/dL)	154.7	158.7	125.0	106.0	9.50	<0.01	0.10
Glucose (mg/dL)	243.3	262.7	258.7	253.0	6.66	0.92	0.08
Albumin (g/dL)	1.33	1.40	1.33	0.93	0.08	0.59	0.28

^a^Data are least squares means of 6 observations per treatments

^b^Basal diet was supplemented with RBO at 5, 10, or 20 g/kg

^c^Pooled standard error of the means

**Table 6:** Immune response of broiler chickens fed the diet containing rice bran oil (RBO)^a^

Items	Dietary treatments^b^				Pooled SEM^c^	*P*-value	
	0	5	10	20		Linear	Quadratic
IgG (mg/mL)	1.26	1.56	1.56	2.51	0.51	0.01	0.27
IgM (μg/mL)	132.2	259.7	162.6	225.9	82.18	0.63	0.71

^a^Data are least squares means of 6 observations per treatments

^b^Basal diet was supplemented with RBO at 5, 10, or 20 g/kg

^c^Pooled standard error of the means

## Conclusion

The results of this study indicate that dietary supplementation of rice bran extracts improves growth performance and decreases total cholesterol level in broilers. Therefore, dietary rice bran extracts is considered a valuable functional ingredient to improve the growth performance of birds.
